# Multicenter Retrospective Analysis of Implant Overdentures Delivered with Different Design and Attachment Systems: Results Between One and 17 Years of Follow-Up

**DOI:** 10.3390/dj6040071

**Published:** 2018-12-11

**Authors:** Marco Tallarico, Luca Ortensi, Matteo Martinolli, Alessio Casucci, Emiliano Ferrari, Giuliano Malaguti, Marco Montanari, Roberto Scrascia, Gabriele Vaccaro, Pietro Venezia, Erta Xhanari, Ruggero Rodriguez y Baena

**Affiliations:** 1 Implantology and Prosthetic Aspects, Master of Science in Dentistry Program, Aldent University, 1001 Tirana, Albnia; ertaxhanari@hotmail.com; 2 Private Practice, 00151 Roma, Italy; 3 Private Practice, 40123 Bologna, Italy; lucaortensi15@gmail.com (L.O.); emo72@tiscali.it (E.F.); gabriele_vaccaro@yahoo.it (G.V.); 4 Private Practice, 45014 Porto Viro, Italy; matteo.martinolli@hotmail.it; 5 Private Practice, 53045 Montepulciano Stazione, Italy; alessio.casucci@gmail.com; 6 Private Practice, 41121 Modena, Italy; malagutig@libero.it; 7 Private Practice, 41121 Cesena, Italy; montmarco@virgilio.it; 8 Private Practice, 74121 Taranto, Italy; roberto.scrascia@gmail.com; 9 Department of Prosthodontics, University of Catania, 95124 Catania, Italy; pierovenezia@gmail.com; 10 Department of Clinical, Surgical, Diagnostic and Pediatric Sciences, School of Dentistry, University of Pavia, 27100 Pavia, Italy; ruggero.rodriguez@unipv.it

**Keywords:** Implant, overdenture, prosthesis

## Abstract

Purpose: To analyze implant and prosthetic survival rates, complications, patient satisfaction, and biological parameters of patients rehabilitated with implant overdentures (IOV) on splinted and nonsplinted implants and different attachment systems, in function for one to 17 years. Methods: This retrospective study evaluated data collected from patients rehabilitated with implant overdentures between January 2001 and December 2016 in nine different centers. Outcome measures were implant and prosthetic success rates, mechanical complications, marginal bone loss (MBL), oral health impact profile (OHIP), bleeding on probing, and plaque index. Results: A total of 581 implants were installed in 194 patients. Patients were followed for a mean period of 60.6 months (range 6–206). Eighty-nine patients received 296 low profile attachment (OT Equator), 62 patients received 124 ball attachments, and 43 patients received 107 Locator attachments. In eighty-three patients the implants were splinted with computer aided design/computer aided manufacturing (CAD/CAM) or casted bar. At the last follow-up, 10 implants failed in eight patients. Statistical significance was found for failed prostheses (P = 0.0723) and complications (P = 0.0165), with better values for splinted implants. No statistically significant differences were found in proportion of implant and prosthetic failure (P > 0.05). At a five-year follow-up, proportion of complications (P = 0.0289) and failed prostheses (P = 0.0069) were statistically higher for IOV on Locator attachments. No difference was founded in MBL at one- and two-year follow-up between different attachment systems (P > 0.05). Statistically significant improvement in all the OHIP categories was reported in all the patients, after one year of function. Conclusions: Implant overdenture showed high implant and prosthetic survival rates, low complications, high patient satisfaction, and good biological parameters in the long-term follow-up. Splinting the implants may reduce number of mechanical complications. Locator attachments showed higher number of complications. Further studies are needed to confirm these preliminary results.

## 1. Introduction

Edentulism can be associated to significant functional impairment as well as unfavorable aesthetic and psychological changes in patients. Problems related to edentulism incorporate limitations in diet and reduced ability to eat certain foods [1,2,3], speech impairment, loss of support for facial musculature, and decreased vertical dimension [4]. Furthermore, edentulism has been defined from The World Health Organization as a physical handicap [5].

For decades, complete removable denture was the conventional solution for treating edentulism. However, conventional complete denture (CCD) can restore chewing function only partially [6]. Furthermore, complete edentulism has an inevitable progressive and irreversible process of basal bone loss [7,8]; leading to augmented difficulties for the denture patient, especially in relation to the mandible [9]. Problems related to increasing basal bone loss include less retention and stability, augmented hyperplasia and ulceration of the underlying mucosa, increased loss of function due to soreness and pain, impaired psychosocial functioning [10] and an augmented risk of choking [11]. To overcome these problems, when a fixed implant-supported prosthesis is not indicated (e.g., excessive inter-arch discrepancy, financial problems, etc.) the use of implant overdentures (IOD) were shown to be successful in rehabilitating the edentulous patients [12,13,14], with high implant success rate [15]. Furthermore, denture stability, masticatory function and patient satisfaction significantly increased when compared with CCDs [16,17,18,19]. According to the glossary of prosthodontic terms, an overdenture is any removable dental prosthesis that covers and rests on one or more remaining natural teeth, the roots of natural teeth, and/or dental implants. Implant overdenture can be implant-retained or implant-supported.

The implant-retained overdenture transfers masticatory forces to the dental implants and to the underlying bone (and the alveolar mucosa). The purpose of the dental implants is to avoid the lateral and vertical dislodgment of the complete denture. An implant-supported overdenture transfers all of the masticatory forces to the dental implants, and as a consequence, to the alveolar and basal bone, such as a fixed solution. This type of prosthesis offers the advantages of being completely supported by implants for increased comfort, but is removed by the patient to maintain proper oral hygiene. Ideally, an implant-supported prosthesis, transferring more load to the implants, requires an increased number of dental implants for its successful outcome compared to an implant-retained prosthesis. However, biomechanics is not the only criterion in the treatment planning of the edentulous patient. Other factors, such as esthetics, speech, cost, ease of maintenance, and patient expectations, play a major role in treatment planning.

Over the years, implant-retained overdentures have been increasingly accepted as an alternative to conventional dentures for oral rehabilitation of edentulous patients; consequently, the type of implant attachment and application methods were diversely developed. The attachment systems for dental implant overdentures can be classified into the self-standing type and bar-type [15]. Self-standing type attachments, such as ball attachment, magnet attachment, and the Locator, have advantages such as easiness in oral hygiene maintenance and possibility of using in a narrow inter-arch space. On the other hand, limits could be found in parallel implant placement requirement, and stability of the implant overdentures lesser to that of bar-type [20,21,22].

Despite several advantages of implant overdenture, biological (e.g., nonosseointegration, mucositis with or without inflammatory hyperplasia, peri-implantitis) and biomechanical complications (e.g., bar fracture, fracture or detachment of the clip anchorage, fracture of the prosthesis or parts of it) can occur during function. Scientific evidence from literature review shows higher frequency of prosthetic complications, particularly for maxillary implant-retained or implant-supported overdentures [20]. On the other hands, short term independent follow-up studies showed lower level of complications with both implant-retained and supported-overdentures [20,21,22,23,24,25]. Furthermore, in most of these study, the reported complications were minor technical issues, resolved chairside. While, the overall incidence of biological complications, such as, peri-implantitis, remain lower due to high level of hygiene maintenance with removable denture compared with fixed solutions. These data are encouraging comparing to biological and technical complications reported with fixed solutions [26].

Recently, computer aided design/computer aided manufacturing (CAD/CAM) titanium bar were developed to fully stabilize an implant-overdenture (“over-implant”) for both mandible and maxilla, showing high oral health-related quality of life and low incidence of complications [23,24].

The aim of this retrospective study is to report implant and prosthesis survival rates, mechanical complications, patient satisfaction, and biological parameters of patients rehabilitated with an implant-retained or -supported overdentures (IODs) on nonsplinted or splinted implants and different attachment systems, in function for a mean period of five years (1 to 17 years).

## 2. Materials and Methods 

A retrospective chart review of existing data, documents, radiographs, and digital files was performed by at each center to evaluate data collected from fully or partially edentulous patients, aged 18 years or older, rehabilitated with an IOD on 1 to 6 implants between January 2001 and December 2016. Data analysis was designed to preserve the anonymity of the patients. Nine expert clinicians performed all surgical and prosthetic procedures in nine private practice centers in Italy. Restorations were delivered by a variety of dental technician in Italy. This study was conducted according to the principles embodied in the Helsinki Declaration of 1975 for biomedical research involving human subjects, as revised in 2000. Patients gave their written informed consent for all the surgical and prosthetic treatments.

Any potential implant locations were considered eligible in this study. Inclusion criteria were: any completely or partially edentulous patients who received an IOD retained or supported by one to six implants with at least 6 months after loading follow-up. Exclusion criteria were: general medical contraindication to oral surgery (American Society of Anesthesiologists, ASA, class III or higher), irradiation in the head and neck area less than one year before implant installation, psychiatric problems, alcohol or drug abuse.

All patients received preoperative photographs, either periapical radiographs or panoramic x-rays, and model casts for initial screening and evaluation. Before implant placement, all of the ptients received a single dose of an antibiotic (2 g of amoxicillin or 600 mg of clindamycin or 500 mg aztromicin if allergic to penicillin). Patients received 1 to 6 implants placed according to the drilling protocol recommended by the manufacturer. A flapless approach was planned in the case of post-extractive implants or in a healed site, depending on the width of the available keratinized mucosa. In cases of bone regeneration, implants were placed six to nine months later. In the case of immediate post-extractive implants, residual teeth were extracted as atraumatically as possible. Implant insertion was then planned along the lingual socket wall, about 1.5 mm below the buccal alveolar crest. The residual socket was grafted with particulated heterologous bone. Loading protocols varied based on implant stability. Nonsubmerged protocol or immediate loading (within 48 h of implant placement) was performed in the case of an implant torque insertion of at least 35 Ncm. After implant placement, all of the patients received oral and written recommendations on medication, oral hygiene maintenance and diet. Postoperative antibiotic therapy (1 g of amoxicillin or 300 mg of clindamycin or 500 mg azytromicin) was 6 and 18 hours after the intervention. Analgesics (500 mg of paracetamol plus 30 mg of codeine, or 600 mg of ibuprofen or 100 mg of nimesulide) were administered as needed. Final restorations were delivered between two to seven months after implant placement.

**Figure 1:**
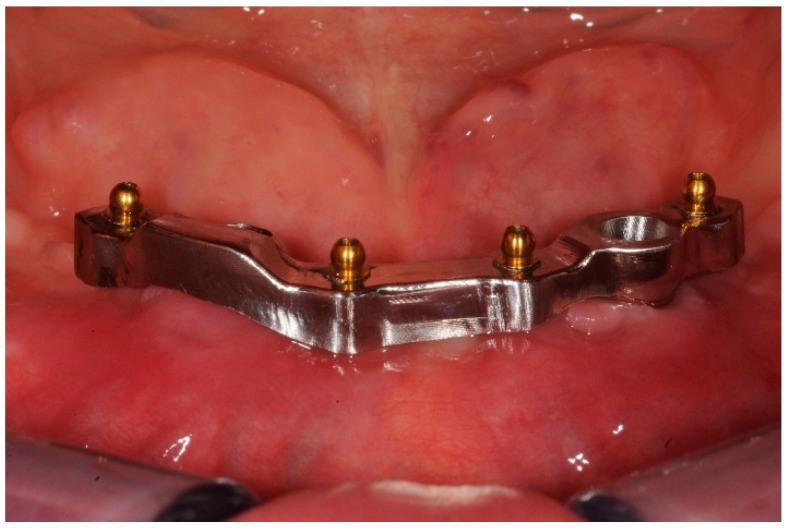
Mandibular overdenture bar with balls attachments.

**Figure 2:**
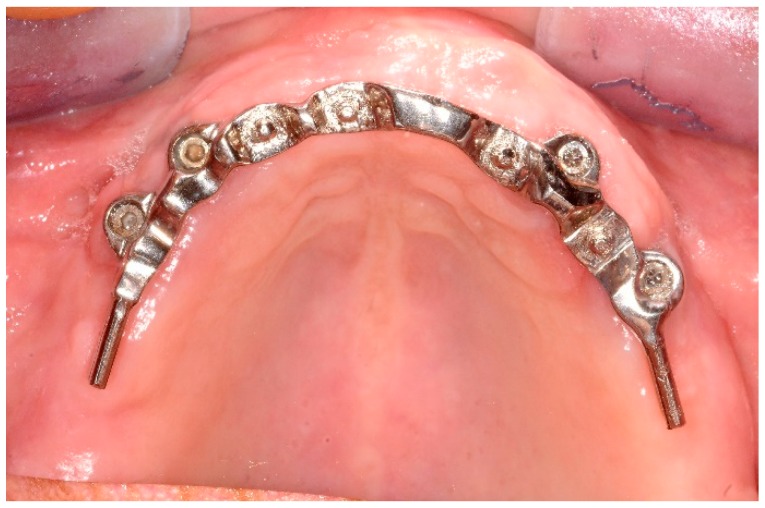
Ten-year follow-up of maxillary overdenture with castable attachments.

**Figure 3:**
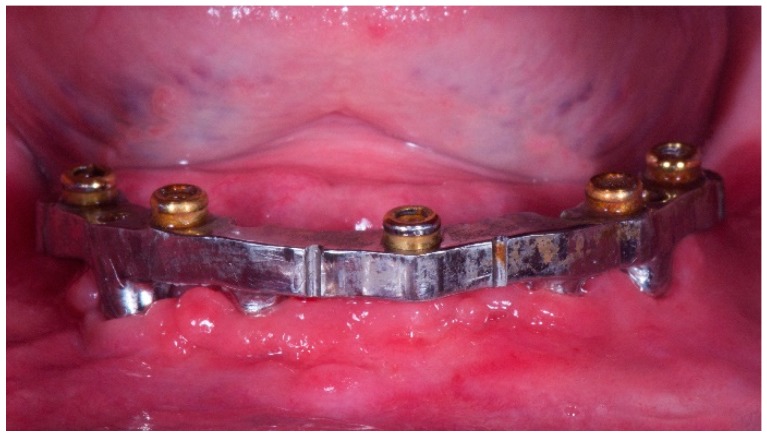
Five-year follow-up of CAD/CAM mandibular overdenture with Locator attachments. Worn attachments in the retentive area, signs of wear due to the rigidity of the attack that makes it work are in the area of maximum circumference.

**Figure 4:**
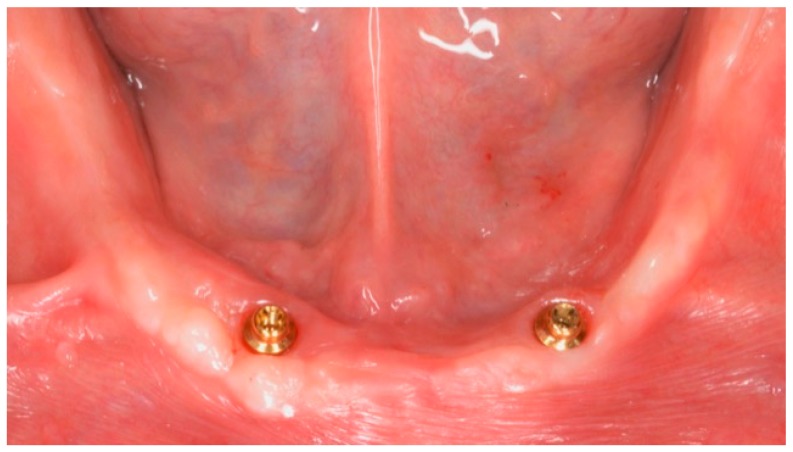
Two-year follow-up of mandibular overdenture with low profile attachments (OT Equator).

Patients were rehabilitated with both implant-retained or -supported overdentures. In case of implant-retained overdentures, pre-existing or a new developed complete removable dentures were used followed standardized techniques [25]. Implant-retained overdentures were delivered on 1 to 5 unsplinted implants. The following attachment systems were used: Equator attachments (OT Equator, Rhein83, Bologna, Italy), ball attachments (OT Cap, Rhein83), or Locator attachments (Zest Dental Solutions, Carlsbad, CA, USA) (Figure 1, Figure 2, Figure 3 and Figure 4). The Rhein83 OT Equator is a low profile castable and direct implant overdenture attachments with a low vertical profile of 2.1 mm and diameter of 4.4 mm. This system offers multiple solutions for overdenture treatment planning when vertical space limitations are a consideration.

All the attachment systems were incorporated chairside into the fitting surface of the overdenture. All dentures were designed or rebased (if the pre-existing dentures were used) to obtain an optimal mucosa support. Most of dentures were reinforced with a cast cobalt-chromium framework. Denture teeth were set with the lingualized occlusion.

In case of implant-supported overdentures, three to six implants were used. Either conventional melting technique or newly developed CAD/CAM technologies were used to fabricate the implant-bar and the metal counterpart according to a previously published protocol [23,24]. Standardized laboratory procedures were accomplished by various dental technicians, according to a previously published protocol [23,24]. The occlusion was developed to deliver a mutually protected articulation and adjusted to avoid any premature contacts. Follow-up visits were scheduled at one and six months after prostheses delivery and then annually. At every follow-up visit, occlusal adjustment was per-formed if needed, and periapical radiographs with a film holder (Rinn XCP, Dentsply Intl) were made annually. The participants were instructed on daily maintenance hygienic procedures and underwent a professional cleaning by a dental hygienist every four to six months.

## 3. Outcome Measures

An implant was classified as successful when the following criteria were fulfilled: it did not cause pain or suppuration, did not show any mobility, did not show any signs of RX radiolucency, did not show peri-implant bone loss >1.5 mm (first year) and then >0.2 mm (yearly). An implant was classified as surviving when the implant remained in the jaw and was stable after the prosthesis was removed.

A complete implant-retained or supported Fixed Dental Prosthesis (FDP) was defined as successful when the dental prosthesis remained in function and the esthetic evaluation was satisfactory.

Biologic (pain, swelling, or suppuration) and/or technical complications (fracture of the framework and/or the veneering material, screw loosening, or screw and/or implant fracture) were recorded.

Marginal bone loss (MBL) was evaluated yearly on intraoral digital radiographs made with the paralleling technique using a film holder (Rinn XCP, Dentsply Intl). All readable radiographs were displayed in an image analysis program (DFW2.8 for Windows; Soredex) that was calibrated for every image using the known pitch of two consecutive implant threads. The radiographs were accepted or rejected for evaluation based on the clarity of the implant threads. Mesial and distal bone level changes were calculated to the nearest 0.01 mm. The patient was used as the statistical unit of the analysis.

The quality of life was assessed by the Oral Health Impact Profile (OHIP-21) questionnaire, which was completed by the participants. The questionnaire consists of seven subscales (functional limitations, physical pain, psychological discomfort, physical disability, psychological disability, social disability, and handicap), with two to four questions each. Participants chose among five possible responses for each question as follows: never, hardly ever, occasionally, fairly often, and very often. Items were scored on a five-point, ordinal-type scale ranging from 1 (never) to 5 (very often). Lower OHIP total scores are suggestive of improvement in oral health-related quality of life (OHRQoL). The questionnaire was administered before treatment and then yearly.

Soft tissue parameters around the implant/abutment interfaces were assessed yearly with a plastic periodontal probe (Plast-o-Probe; Dentsply Maillefer). The bleeding index (BI) was evaluated at 4 sites around each implant according to the Mombelli index, and the plaque index (PI) was evaluated for each implant according to the same author.

Patient data were collected in a spreadsheet (Numbers Version 3.6.1 for Mac OS X 10.11.4). A biostatistician with expertise in dentistry analyzed the data using SPSS software for Mac OS X (version 22.0; SPSS Inc., Chicago, IL, USA) for statistical analysis. Descriptive analysis was performed for numeric parameters using mean ± standard deviation with confidence interval (95% CI). Median and interquartile (IRQ) values were also calculated for bleeding on probing and plaque index in order to give a better description of our data set. Analysis of the ten-year cumulative implant survival rate (CSR) was performed at patient level, according to the life table method and illustrated with Kaplan–Meier survival curves. Differences in the proportion of patients with implant failures, prosthesis failures and complications (dichotomous outcomes) were compared between using the Fisher’s exact probability test and the Risk Ratio (.95 Confidence Interval). Differences of means at patient level for continuous outcomes (OHIP, marginal bone loss, BoP and PI) were compared by independent sample *t* tests. All statistical comparisons were conducted at a 0.05 level of significance.

## 4. Results

Nine centers reported data from 581 implants, installed in 194 patients (120 females and 74 males; mean age 68.6 years, range 39–90) that received one to six implants. Five patients received only one implant; 92 patients received two implants each; 15 patients received three implants each; seven patients received four implants each; five patients received five implants each; and seven patients received six implants each (Table 1).

Thirty-three patients used to smoke more than 10 cigarettes per day (17.0%). Patients were followed for a mean period of 60.6 months (range 6–206). Sixty-nine patients (35.6%) with 249 implants (42.9%) were rehabilitated in the maxilla; while, 125 patients (64.4%) with 332 implants (57.1%) were rehabilitated in the mandible. Eight-nine patients received 296 OT Equator attachments (Rhein83), 62 patients received 124 ball attachments, and 43 patients received 107 Locator attachments (Zest). In 83 patients the implants were splinted (29 CAD/CAM titanium bar and 54 Cromo Cobaltium bar made with conventional lost wax technique), while, in 111 patients the implants were unsplinted (Table 2). Of these, 72 implant-retained overdentures were metal-reinforced. Most of the splinted implant-supported overdentures was designed with only one bar (one-piece, n = 79) while four metal bars were two-piece. The counterpart was made with conventional lost wax casting technique in 62 patients, while, in 21 patients the counterpart was made using laser melting technique. In 60 patients, the veneering material was composite, in 130 was resin, and in four patients was ceramic. Most of the patients (n = 116) were mesocephalic, while 42 were brachycephalic, and 23 dolichocephalic. In 13 patients data on facial type was not available. Patients and implants characteristics were reported in Table 3, Table 4, Table 5 and Table 6.

Two biological complications were experienced in one smoking patient, resulting in 3 mm of bone loss at the one year of follow-up examination. The patient was enrolled in a strictly hygiene maintenance program with visit every four months, and no further pathological bone loss was experienced. No major biological complications were experienced, such as implant suppuration or mobility.

At the one-year follow-up examination (194 patients with 581 implants), seven implants (1.2%) in five patients (2.6%) failed. All the implants failed in the maxilla in class VI of Cawood and Howell patients (P = 0.0002). Only one prosthesis failed (0.5%). Fourteen complications were experienced in 13 patients (6.7%). At the two-year follow-up examination (126 patients with 385 implants), one implant (0.3%) in one patient (0.8%) failed. One prosthesis failed (0.8%). Four complications were experienced in 4 patients (3.2%). At the three-year follow-up examination (103 patients with 218 implants), one implant (0.5%) in one patient (1.0%) failed. Two prostheses failed (1.9%). Two complications were experienced in two patients (1.9%). At the five-year follow-up examination (61 patients with 181 implants), one implant failed (0.6%) in one patient (1.6%). One prosthesis failed (1.6%). Five complications were experienced in five patients (8.2%).

Overall, 10 implants (1.7%) failed in eight patients (4.1%). No statistically significant differences were found between failure in the maxilla and mandible (7/242 versus 3/329; P = 0.1079). Of these, 70% of the implant failures were experienced before loading. A total of five prosthesis failed scoring a cumulati survival rate of 97.4%. A total of 25 complications were experienced in 24 patients resulting in a cumulative implant success rate of 87.6%. Most of the complications were experienced in the unsplinted group. There was a statistically significant difference when comparing the overall number of complications and the number of the placed implants to retain an implant overdenture, with higher value when four implants were used (P = 0.000465).

At the five-year follow-up examination, no statistically significant difference was found for failed implants between splinted and unsplinted designs (4/79 versus 4/107; P = 0.7261; RR = 0.9874; 0.9296–1.0488). Statistically significant difference was found for failed prostheses (0/83 versus 5/106; P = 0.0723; RR = 1.0472; 1.005–1.09037) and complications (5/78 versus 20/91; P = 0.0165; RR = 1.1463; 1.0343–1.2704), with better values for splinted implants. No statistically significant differences were found between overdentures delivered on splinted and unsplinted implants for OHIP, MBL, BoP, PI at each time points, up to five years on function (P ≥ 0.05).

Within the unsplinted group, at the five-year follow-up examination, no statistically significant difference was found for failed implants (1/71 versus 0/39; P = 1.0; RR = 0.9861; 0.9594–1.0135); failed prostheses (5/67 versus 0/39; P = 0.1597; RR = 0.9306; 0.8737–0.9912) and complications (9/63 versus 5/34; P = 1.0; RR = 1.0037; 0.865–1.1646), between metal-reinforced or not reinforced IODs.

At the last follow-up examination, no statistically significant difference was found for failed implants between different attachment systems (OT Equator, Rhein83; OT Cap, Rhein83; and Locator, Zest Dental Solutions) with respectively 5/84; 2/60; and 3/40 (P = 0.6487). On the contrary, statistically significant difference was found for failed prostheses (0/89; 1/61; and 4/39; P = 0.0069) and complications (6/83; 8/54; and 10/33; P = 0.0289), with better values for OT Equators (Rhein83) attachment systems.

Data from MBL between different attachment systems were available at the one- and two-year follow-up examination. No statistically significant differences were found between groups. At the one-year follow-up examination, the mean MBL was 0.32 mm; 0.24 mm; and 0.29 mm for OT Equator (n = 60, Rhein83), OT Cap (n = 56, Rhein83) and Locator, (n = 26, Zest Dental Solutions), respectively (P = 0.4640). At two-year follow-up examination, the mean MBL was 0.36 mm; 0.34 mm; and 0.36 mm for OT Equator (n = 36, Rhein83), OT Cap (n = 50, Rhein83) and Locator, (n = 19, Zest Dental Solutions), respectively (P = 0.062).

One-year after loading (n = 142), the mean marginal bone loss was 0.28 ± 0.43 mm (0.11–0.26). Two years after loafing (n = 105), the mean marginal bone loss was 0.35 ± 0.54 mm (0.07–0.27). The difference from the previous follow-up was not statistically significant (P = 0.2602). Three years after loading (n = 67), the mean marginal bone loss was 0.38 ± 0.45 mm (0.09–0.31). The difference from the previous follow-up was not statistically significant (P = 0.7386). Five years after loading (n = 46), the mean marginal bone loss was 0.46 ± 0.41 mm (0.16–0.39). The difference from the previous follow-up was not statistically significant (P = 0.3535). No differences were found in MBL between different facial types at each follow-up (Table 7).

The OHIP questionnaire were delivered in 162 patients. Before treatment (baseline), mean OHIP was 73.1 ± 9.4 (71.1–74.0). One year after loading, 153 patients answered to the questionnaire. The mean OHIP was 26.8 ± 9.1 (22.6–25.5). The difference was statistically significant (P = 0.0000). Two years after loading OHIP values were available from 109 patients. The mean value was 28.1 ± 8.7 (23.9–27.1). The difference from baseline was statistically significant (P = 0.0000). Three years after loading OHIP values were available from 71 patients. The mean value was 31.1 ± 7.9 (27.1–30.8). The difference from baseline was statistically significant (P = 0.0000). At the 5-year follow-up examination, 55 patients answered the questionnaire. The mean OHIP was 32.4 ± 7.2 (28.6–32.4). The difference from baseline was statistically significant (P = 0.0000).

One-year after loading (n = 153), the mean bleeding on probing was 0.07 ± 0.10 mm (0.00; 0.02; 0.13). Two years after loading (n = 106), the mean bleeding on probing was 0.11 ± 0.17 mm (0.00; 0.06; 0.13). The difference from the previous follow-up was statistically significant (P = 0.0460). Three years after loading (n = 70), the mean bleeding on probing was 0.09 ± 0.18 mm (0.00; 0.00; 0.13). The difference from the previous follow-up was not statistically significant (P = 0.5639). Five years after loading (n = 45), the mean bleeding on probing was 0.10 ± 0.17 mm (0.00; 0.00; 0.13). The difference from the previous follow-up was not statistically significant (P = 0.9361).

One-year after loading (n = 151), the mean plaque index was 0.12 ± 0.15 mm (0.00; 0.05; 0.25). Two years after loafing (n = 106), the mean plaque index was 0.12 ± 0.17 mm (0.00; 0.00; 0.20). The difference from the previous follow-up was not statistically significant (P = 0.9957). Three years after loading (n = 70), the mean plaque index was 0.12 ± 0.17 mm (0.00; 0.00; 0.22). The difference from the previous follow-up was not statistically significant (P = 0.9986). Five years after loading (n = 45), the mean marginal bone loss was 0.09 ± 0.16 mm (0.00; 0.00; 0.13). The difference from the previous follow-up was not statistically significant (P = 0.3378).

## 5. Discussion

This retrospective study evaluated long-term implant and prosthetic success rates, mechanical complications, oral health impact profile, marginal bone loss, bleeding on probing and plaque index of 581 implants placed on 194 patients to delivery implant-retained or -supported overdentures and followed for up to 17 years in function.

The main limitation of this study is its retrospective nature. As such, only a few cases (29.4%) were within the five to 17 years cohort, the clinician should interpret with caution data emerged in this paper. Moreover, thickness of the mucosa was not evaluated in this research. However, the relatively high overall number of implants and patients, as well as the relatively long period of follow-up may provide important insight helpful in a daily practice.

According literature data [15], in the present study, the implant cumulative survival rate was 95.9% at patient level, after 17 years of loading, with an average follow up of five years. Furthermore, according to Awad et al. 2000 [27], high patient satisfaction where reported during the follow-up, mainly due to improved denture stability and masticatory function.

Although well-established results are reported in the international literature, there are no specific guidelines or consensus regarding the number of implants needed to delivery an implant-retained or -supported overdenture. In clinical practice, four endosseous implants are considered the minimum number needed for maxillary overdenture treatment, while only two for the mandibular overdenture, as determined based on survival rate studies [28,29,30,31,32]. In the present study, although no statistically significant difference was reached, implants placed in the maxilla fail three times more that implants installed in the mandible, particularly in Cawood and Howell class VI patients. Accordingly, most of the overdenture in the mandible were delivered on two implants (86.4%) while in the maxilla, 59.1% of the prosthesis were delivered on four implants. Nevertheless, even in Cawood and Howell class VI patients, an implant-supported overdenture on four implants seem to be the gold standard to reduce implant failure.

In the present study, 111 patients received an implant retained-overdenture delivered on unsplinted implants, while in 83 patients the implants were splinted by means of 29 CAD/CAM titanium bar and 54 Cr-Cb bar made with conventional melting technique. Even if a splinted design has been considered a more reliable option [33,34,35], authors found no statistical differences between overdenture on splinted and not splinted implants for implant failure, OHIP, MBL, BoP, PI at each point in time, up to five years on function (P ≥ 0.05). Nevertheless, prosthetic failure and complications were statistically lower in the splinted group. Conversely, Slot et al. [34] in a systematic review of maxillary overdentures, found a survival rate of 98.2% in case of six implants and a bar anchor-age, a survival rate of 96.3% in case of four implants and a bar anchorage, and a survival rate of 95.2% in case of four unsplinted implants with ball attachment system, after one year of treatment.

Nowadays, implant-retained or -supported overdentures can be considered a viable treatment option when bone volume is reduced. The IODs increase the masticatory function and improve satisfaction by making up for insufficient retention and stability of a conventional denture [28].

In the present study, when splinted implants were used to support a maxillary or mandibular implant overdenture, less complications were experienced compared to conventional implant-retained overdenture on unsplinted implants, independently by the presence of a metal reinforcement. A probable motivation should be that the occlusal forces were distributed onto the connected implants and the metallic bar, also requiring an increased number of implants for its successful outcomes [36,37,38]. Also in a five-year prospective study of Krennmair et al. less prosthodontic maintenance, i.e., for clip activation/fracture, was referred when four interforaminal splinted implants were used to support a mandibular overdenture [38].

The present study failed to found statistically significant differences related to different facial type, in any of the investigated outcomes (P > 0.05). In a study of Ahmad et al. [39] the gonial angle was found to be significantly correlated with residual ridge resorption associated with implant-retained overdentures. Although in the present study no differences were found, a trend of higher marginal bone loss was found in brachyfacial individuals. In these patients, an implant-supported overdenture could be a valid treatment option to prevent biological complication associated with higher marginal bone loss.

In the present study, Locator attachments showed higher number of complications and prosthetic failure. These results are in agreement with the research of Krennmair at al. that reported more post insertion aftercare (activation of retention) for Locator attachment compared to the ball anchors [37,38]. A possible explanation for this results could be that the shape of the OT Equator retained system seems to collect minor stress around the periimplant bone tissue and the fixture itself [40]. Nevertheless, confounding factors, such as the number of implants could influenced the results. In fact, when standard commercial attachments are used dangerous occlusal forces can be partially distributed on mucosa only under denture retained on one or two implants, and the axial mobility cannot help in increase of use of mucosal supporting, reducing implant loading [41,42]. In the present study, statistically significant differences in complications were found when four implants are used to retain an implant overdenture.

Overall, in the present study, five prostheses have to be remade, while 25 complications were experienced in 24 patients. Most of them were tooth or matrix detachment resolved, chairside in less than 60 minutes. According to the international literature, the few studies that mentioned aspects of prosthetic aftercare provided to implant-retained maxillary overdentures reported complications with the attachment components [43], fractures of the acrylic resin or teeth [44,45], or overdenture adjustments [43].

Five years after loading the mean marginal bone loss was 0.46 ± 0.41 mm, with a minimum of 0.12 mm and a maximum of 2.13 mm. It might be noticed that these results may be in line or even better of the mean marginal bone loss reported by Meijer et al. 2014 [46]. The authors report a mean MBL of 1.0 and 1.1 mm with implant-retained overdenture in function for 5 and 10 years respectively. According to the results of a systematic review of Cehreli et al., in the present study there was no statistically significant difference between splinted or unsplinted implants as well as between different type of attachment systems [47].

It is widely accepted that conventional complete removable denture has less satisfaction results in patients life compared to IODs [12,18,19,48,49]. In the present study, statistically significant improvement in all the OHIP categories was reported in all the patients, after one year of function. No differences were found for different IODs design or attachment systems.

Plaque scores increased slightly during function independently by the number of the implants and the type of anchorage. Elsyad MA et al. reported that this increase in plaque scores could be associated to the resiliency of both attachments, which allow denture movements and accumulation of plaque under the denture [50]. Also age related problems as decreased awareness could affect oral hygiene practice of the patients [49].

The elastic material of the retentive matrix of OT Equator may allow to distribute on a larger surface the retentive capacity, resulting in a longer lasting retention due to the wear reduction at the circumference.

The rigid attachments such as the Locators only work on the circumference and have very thin rigid material matrices.

It should be noticed that the retentive force of the Locator and OT Equator attachments is obtained through mechanical interlocking and frictional contact between the male and female. An ideal attachment system should provide a high and stable retentive force with a low lateral force to the implant, not only in the parallel placement of the implant, but also in the implant inclination during recurrent dislodging [51]. The retention feature of the Locator and OT Equator attachments is a frictional contact, which derives from a dimensional misfit between the slightly oversized male and the smaller diameter of the female abutment.

Both attachments investigated in this paper had the same clinical advantages, nevertheless, a less number of complications and prosthetic failure can be expected using OT Equator. A possible explanation could be that the retentive caps of the OT equator are made of elastic material while Locator uses rigid material. Elastic material seems to work better than rigid. Furthermore, the smaller size of the OT Equator may allow for an improved design of the overdenture leaving more space for the veneering materials. Furthermore, by exploiting the low profile of the OT Equator the clinician can better manage the prosthetic spaces according to a better aesthetic result.

## 6. Conclusions

Implant overdenture showed high implant and prosthetic survival rates, low complications, high patient satisfaction, and good biological parameters in the long-term follow-up. Splinting the implants may reduce number of complications. Locator attachments showed higher number of complications. Further studies are needed to confirm these preliminary results.

## Figures and Tables

**Table 1:** Implant distribution.

Number of Implants	1 Implant	2 Implants	3 Implants	4 Implants	5 Implants	6 Implants
Number of patients	5	92	15	70	5	7

**Table 2:** Attachment distribution.

Attachment	OT Equator	Ball Attachments	Locator
Number of patients	89 (296)	62 (124)	43 (107)

**Table 3:** Patients and implants characteristics according to the different centers.

Centre	Patients (Implants)	Mean Follow-Up	Maxilla	Splinted	Unsplinted	Failed Implants Last FU	Failed Prosthesis Last FU	Complications Last FU
MT	19 (62)	22 (12–48)	6	10	9	0	0	2
AC	14 (30)	17.1 (12–24)	0	0	14	1	0	4
MM	19 (70)	28 (8–44)	13	14	5	4	0	1
RS	28 (72)	30.4 (12–74)	7	4	24	0	3	2
PV	8 (20)	19.5 (12–36)	1	2	6	0	0	1
EF	9 (34)	31.1 (12–54)	6	7	2	1	0	2
LO	66 (176)	104 (6–206)	26	26	40	1	2	7
GM	18 (76)	49 (12–88)	7	18	0	0	0	0
GV	13 (41)	79.5 (13–150)	3	2	11	3	0	6
Total	194 (581)	60.6 (6–206)	69	83	111	10	5	25

**Table 4:** Implants outcomes according to the different location (maxilla or mandible).

Location	Patients (Implants)	Implant	Splinted	Unsplinted	Failed Implants Last FU	Failed Prosthesis Last FU	Complications Last FU
Maxilla	69	249	47	22	7	2	11
Mandible	125	332	36	89	3	3	14
Total	194	581	83	111	10	5	25
P Value					0.0360	1.000	0.3752

**Table 5:** Patients distribution according to the Cawood and Howell classification.

Cawood & Howell Classes	%	Number of Patients
C&H II	16.6	30
C&H III	35.3	64
C&H IV	33.7	61
C&H V	6.1	11
C&H VI	8.1	15

**Table 6:** Patients distribution according to the occlusal scheme.

Occlusal scheme	%	Number of Patients
Anterior	5.7	11
Group function	39.2	76
Bilateral	55.1	107

**Table 7:** MBL (mm) between different facial types.

Facial Type	1 Year	2 Years	3 Years	5 Years
Brachyfacial	0.33 ± 0.45 (n = 38)	0.43 ± 0.54 (n = 34)	0.5 ± 0.56 (n = 24)	0.45 ± 0.26 (n = 13)
Dolicofacial	0.17 ± 0.1 (n = 19)	0.17 ± 0.13 (n = 12)	0.23 ± 0.26 (n = 8)	0.37 ± 0.34 (n = 5)
Mesofacial	0.23 ± 0.23 (n = 84)	0.25 ± 0.29 (n = 59)	0.33 ± 0.40 (n = 35)	0.47 ± 0.47 (n = 28)
	P = 0.1063	0.0534	P = 0.2368	P = 0.8821
